# Reliability and validity of the positive mental health literacy scale in Chinese adolescents

**DOI:** 10.3389/fpsyg.2023.1150293

**Published:** 2023-04-24

**Authors:** Zhanfang Liu, Fangru Yuan, Jin Zhao, Jianzheng Du

**Affiliations:** ^1^School of Education, Guangzhou University, Guangzhou, China; ^2^Xiangnan Preschool Education College, Chenzhou, China; ^3^The First High School of Chenzhou (North Campus), Chenzhou, China; ^4^School of Education Science, Liaocheng University, Liaocheng, China

**Keywords:** mental health literacy, measure, health promotion, reliability and validity, adolescent

## Abstract

**Background and aim:**

Mental health literacy (MHL) is not only the necessary knowledge and ability to promote mental health, but also an important determinant of mental health. Traditionally, the MHL Scale focuses on measuring knowledge and beliefs about mental disorders. In China, there are very few scales for assessing positive MHL. The present study aimed to evaluate the reliability and validity of a Chinese version of the Positive MHL Scale (MHPK-10) in Chinese adolescents.

**Methods:**

Chinese adolescents (*n* = 1,247) completed the MHPK-10 online. The validation included the translation and cultural adaptation of the MHPK-10 original version into Chinese and assessment of its psychometric properties: reliability—test–retest and internal consistency, construct validity and criterion validity.

**Results:**

Participant’s mean score on the revised positive MHL scale was 3.75 (SD = 0.69) which was a unidimensional scale. The correlation coefficients between each item and the total score were between 0.639 and 0.753. Scale item loadings ranged between 0.635 and 0.760 based on confirmatory factor analysis. Cronbach’s α coefficient of the scale was 0.869, and the test–retest intraclass correlation coefficient was 0.721 (*p* < 0.01). Criterion validity was assessed by comparing results of the revised MHPK-10 against those of other validated scales and resulting correlations ranged between 0.342 and 0.615.

**Conclusion:**

The revised Chinese version of the MHPK-10 has sound reliability and validity and can be used to measure Chinese adolescents’ positive MHL.

## 1. Introduction

Around one-third of people worldwide develop a mental disorder at some point in their life (Steel et al., 2014), and the onset of many mental disorders occurs in childhood or adolescence (Carvalho et al., 2022). Mental disorders seriously affect adolescents’ mental health, wellbeing, academic performance and social relations (Campbell et al., 2022) but very few seek professional help (Seo et al., 2022) while others postpone obtaining support (Jorm, 2012). Yet, delaying treatment makes treatment more difficult (de Diego-Adeliño et al., 2010). Patients are often reluctant to seek help because they are not aware that they have a mental disorder (poor disease recognition ability) or are concerned about stigma (Jorm, 2012). Negative public perceptions of mental health issues and professional treatment are manifestations of poor mental health literacy (MHL) (Daehn et al., 2022).

Mental health literacy is considered to be an important factor in promoting an individual’s mental health and may be beneficial to individual and public mental health (Kutcher et al., 2015; Wei et al., 2015; Ming and Chen, 2020). MHL was originally proposed by Jorm et al. (1997) based on health literacy, which refers to “knowledge and beliefs about mental disorders which aid their recognition, management or prevention”. This definition is often considered the “gold standard” for MHL (Spiker and Hammer, 2019). Research on MHL has further refined the definition, to include four components: “(1) understanding how to obtain and maintain good mental health, (2) understanding mental disorders and their treatments, (3) decreasing stigma related to mental disorders, and (4) enhancing help-seeking efficacy (knowing when, where, and how to obtain good mental healthcare and developing competencies needed for self-care)” (Kutcher et al., 2016).

The proposal of MHL has promoted the development of MHL evaluation tools (Jorm, 2015), and Jorm’s “vignettes interview” (Jorm et al., 1997), Connor’s Mental Health Literacy Scale (MHLS) (O’Connor and Casey, 2015) are examples of typical assessment tools (Jiang et al., 2020). However, all measure the mental illness component of MHL only, and not the mental health component. Based on the basic psychological needs theory (BPNT), Bjørnsen et al. (2017) developed the Mental Health-Promoting Knowledge Scale (MHPK-10) to evaluate positive MHL. The emergence of MHPK-10 is an improvement of MHL assessment tools and has been considered to have a positive impact on mental health promotion activities and assessment (Bjørnsen et al., 2017). Pote and Fulcher (2021) evaluated many current MHL scales and believed that the psychological properties of MHPK-10 were sound, and suggested that other researchers use it. At present, revised versions of MHPK-10 have appeared in Portuguese (Guimarães et al., 2022) and Turkish (Özpulat et al., 2022).

Until now, China does not have a scale for evaluating positive MHL. Because MHPK-10 is a tool for evaluating “how to obtain and maintain good mental health” in MHL, can be completed quickly (there are only 10 questions), is easy to understand (Guimarães et al., 2022) and each item can be translated into practice (Bjørnsen et al., 2017), we have chosen to revise the MHPK-10 into Chinese. The problems to be addressed in this study were: (1) To create a culturally appropriate adaptation of MHPK-10 for a Chinese audience. (2) Evaluate the reliability and validity of a Chinese version of the MHPK-10 in Chinese adolescents, and (3) Explain the dimension values compared to the English version (MHPK-10).

## 2. Materials and methods

### 2.1. Participants

#### 2.1.1. Sample 1

**FIGURE 1:**
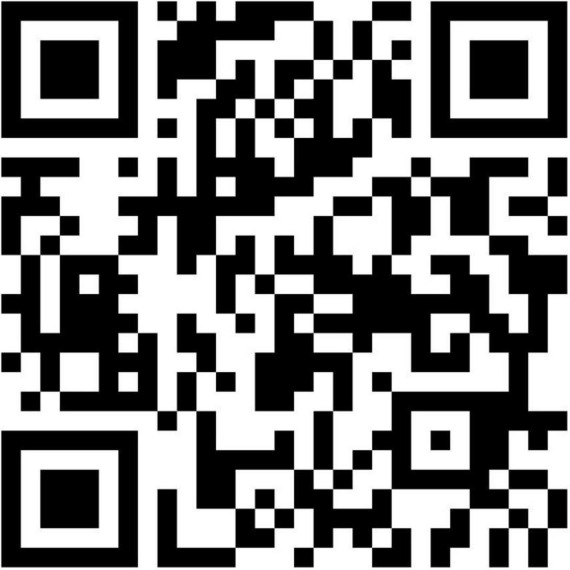
The two-dimensional code.

Over a 2-week period in October 2022, a cross sectional classroom survey was conducted at a junior college in Hunan Province, China. The online questionnaire was prepared using Sojump^1^ software, and the researchers asked subjects to scan a two-dimensional code (see Figure 1) and complete it. A total of 1,382 questionnaires were collected, and questionnaires were deemed invalid if the response time was very short (less than 300 s) or if a participant had answered all items in the same way (or adopted a very regular pattern of responses) throughout indicating that he/she had not read the items or not considered each item carefully (Wu et al., 2020). Finally, 1,247 valid questionnaires were retained, yielding a response rate of 90.23%. The valid questionnaire included 297 male and 950 female; the age ranged from 15 to 22 years, with an average of 17.82 ± 1.61 years. The data of sample 1 were randomly divided into two groups. The data of group 1 (*n* = 609) were used for item analysis and exploratory factor analysis; Group 2’s data (*n* = 638) were used for confirmatory factor analysis, internal consistency and criterion-related validity analysis.

#### 2.1.2. Sample 2

After an interval of about 2 weeks, 103 participants were randomly selected from sample 1 and asked to complete the MHPK-10 a second time to assess its test–retest reliability. After matching by student number and eliminating repeated questionnaires (some people completed the questionnaire more than once), 96 valid questionnaires were matched, including 19 male and 77 female, aged between 16 and 21 years, with an average age of 18.47 ± 1.81 years.

### 2.2. Instruments

#### 2.2.1. Chinese version of positive mental health literacy scale (MHPK-10-C)

The MHPK-10 has 10 items and one dimension. Each item has six answer options and uses a five-point Likert scale where 1 = completely wrong, and 5 = completely correct and “don’t know” (0 when scoring). The final scale score is obtained by averaging the scores of the 10 questions with higher scores indicating higher positive MHL (Bjørnsen et al., 2017).

The MHPK-10 Chinese revision process was as follows: (1) The authorization of the original author was obtained, (2) Three psychologists (2 male, 1 female) established a translation team to translate all items of MHPK-10 individually. (3) Items 8 and 10 were found difficult to understand in the translation process, so we sought the help of the original author and created the first Chinese version of MHPK-10 after receiving feedback. The original author’s feedback was: “Item 8 concerns being able to set limits for yourself, e.g., deciding if you want to drink alcohol based on your own will, or limiting one’s own behavior to what one finds ok. Item 10 concerns the feeling of being able to handle school, and experiencing to some extent that one masters school and schoolwork.” (4) 7 students (3 boys and 4 girls, aged between 16 and 18) were recruited to test the comprehensibility and fluency of each item of the first Chinese version of MHPK-10, and each item was presented individually. (5) After receiving feedback, the translation team revised the first edition of MHPK-10-C to form the second edition. (6) Two English college teachers (both female) back-translated the second Chinese version into English. The original English version, the second Chinese version and the translated English version were back-translated into English, and revised repeatedly, finally forming MHPK-10-C.

#### 2.2.2. Additional measures

The MHPK-10 is an assessment of individual’s positive MHL, and MHL is closely related to mental health. Therefore, we chose positive psychology, MHL and mental health scales to perform a preliminary analysis of criterion validity, as follows:

The short version of the Warwick Edinburgh Mental Wellbeing Scale (SWEMWBS), includes seven items that are responded to using a five-point Likert scale where 1 = none of the time, and 5 = all of the time. Higher scores reflect higher subjective wellbeing (Ringdal et al., 2018; Zhao et al., 2019). In this study, Cronbach’s α was 0.882.

The Mental Health Literacy Questionnaire (MHLQ) developed by Wu et al. (2020) assesses six factors including knowledge of mental health, knowledge of mental illness, attitudes and habits of maintaining and promoting one’s own mental health, attitudes and habits of coping with one’s own mental illness, attitudes and habits of maintaining and promoting others’ mental health, and attitudes and habits of coping with others’ mental illness. The MHLQ is comprised of 60 questions: questions 1 to 30 are scored with either a 0 or 1 (YES = 1, NO = 0, DONT KNOW = 0), and questions 31–60 are scored using a five-point Likert scale, which are converted into 0 and 1 (ratings of 4 and above were re-coded as 1 for positively worded items) points when calculating the total score. The maximum total score is 60, with higher scores indicating better MHL. The internal consistency reliability of the six dimensions ranged from 0.640 to 0.706, and the test–retest reliability of the total score was 0.720 at an interval of 3 weeks. The convergent validity of MHLQ and MHLS was 0.740.

The General Wellbeing Scale (GWBS) (Xiong and Xu, 2009), which has only one question, “How happy are you in general?,” uses a seven-point Likert scale (1 = very unhappy, 7 = very happy) with higher scores reflecting greater happiness.

The Life Satisfaction Scale (LSS; Yao et al., 1995; He, 2021), has only one question: “How satisfied are you with your life?” and is answered using a seven-point Likert scale (1 = very dissatisfied, 7 = very satisfied), with higher scores indicating better life satisfaction.

Self-rated health was assessed by asking participants one question: “How is your current health?” The higher the score, the higher the level of mental health, and was answered using a five-point Likert scale (1 = very poor, 2 = poor, 3 = neither poor or good, 4 = good 5 = excellent). This item has been previously found to be satisfactory for use among adolescents (Breidablik et al., 2008).

The Depression Anxiety Stress Scale includes 21 items, and is responded to using a four-point Likert scale (1 = did not apply to me at all, 4 = applied to me very much or most of the time), with higher scores reflecting lower levels of mental health (DASS) (Gomez et al., 2014; Lu et al., 2020). In this study, Cronbach’s α was 0.919.

The Loneliness scale has only one item, “Do you ever feel lonely?,” which is answered using a five-point Likert scale (1 = never or almost never, 2 = rarely, 3 = sometimes, 4 = regularly, and 5 = almost all the time). Higher scores indicate higher levels of loneliness (Bjørnsen et al., 2019).

### 2.3. Statistical analyses

Exploratory analysis and factor reliability analysis were performed using SPSS 20.0 (IBM Corp. Released 2011. IBM SPSS Statistics for Windows, Version 20.0. Armonk, NY, USA: IBM Corp.), and confirmatory factor analysis was performed using Amos 23.0. In the analysis, the independent sample *t*-test was used to evaluate item discrimination, and Pearson’s correlation coefficient was used to determine the correlation between variables (correlation between each item and total score, criterion-related validity, test–retest reliability). Statistical significance was accepted as *p* < 0.05.

### 2.4. Ethics statement

The study was approved by the Ethics Committee of the School of Education at Guangzhou University (IRB number: GZHU2020010).

## 3. Results

### 3.1. Item analysis

Group 1’s MHPK-10-C responses were classified into two groups. The extreme group method was used to classify them into a high-scoring and a low-scoring group. Those scoring within the highest 24% of scores became the high group (*n* = 147, Total MHPK-10-C ≥ 43), while those scoring within the bottom 27% became the low group (*n* = 164, Total MHPK-10-C ≤ 33). Results of the independent sample *t*-test showed that there were significant differences in MHPK-10-C scores between the high-scoring group and low-scoring group (*p* < 0.01). The correlation coefficients between each item and the total score were between 0.639 and 0.753 (see Table 1).

**TABLE 1:** MHPK-10-C^#^: item means, standard deviations, total correlations, communality, and factor loadings (*n* = 609).

Item	Mean ± SD	Critical value	Pearson correlations	Communality	Factor loadings
Item_1	3.7 ± 1.04	20.026**	0.687**	0.467	0.684
Item_2	3.72 ± 1.01	21.737**	0.753**	0.577	0.760
Item_3	3.38 ± 1.18	20.725**	0.695**	0.460	0.678
Item_4	3.89 ± 0.93	18.957**	0.720**	0.534	0.731
Item_5	3.94 ± 0.88	17.982**	0.651**	0.434	0.659
Item_6	4.03 ± 0.95	16.734**	0.639**	0.403	0.635
Item_7	3.74 ± 1.02	20.368**	0.715**	0.510	0.714
Item_8	3.99 ± 1.01	18.34**	0.669**	0.441	0.664
Item_9	3.76 ± 1.03	20.605**	0.698**	0.491	0.701
Item_10	3.343 ± 1.01	20.342**	0.718**	0.52	0.721
Total MHPK-10-C^#^	3.75 ± 0.70	46.798**			

**p < 0.01. SD, standard deviation; ^#^MHPK-10-C, positive mental health literacy scale (Chinese version).

### 3.2. Structure validity analysis

#### 3.2.1. Exploratory factor analysis

Exploratory factor analysis (EFA) was conducted to examine the structure of MHPK-10-C using group 1’s data (*n* = 609). The Kaiser-Meyer-Olkin (KMO) value was 0.928, and the result of Bartlett’s spherical test was significant (χ2 = 2199.048, df = 45, *p* < 0.01), indicating that the data were suitable for exploratory factor analysis. EFA was performed using the maximum variance rotation method. Based on an eigenvalue greater than 1, one factor with an eigenvalue greater than 1 was extracted, and the cumulative variance contribution rate was 48.38%. According to the standard of commonalities greater than 0.300 and factor loadings greater than 0.400 (Zhou et al., 2022), all items were retained (see Table 1).

#### 3.2.2. Confirmatory factor analysis

Confirmatory factor analysis was performed to examine the factor structure of MHPK-10-C using group 2’s data (*n* = 638). An analysis of the P-P and Q-Q diagrams showed the data conformed to the standard normal distribution. The factor model, based on the results of the EFA is shown in Table 2. This table shows that confirmatory factor analysis results are within acceptable limits for the MHPK-10-C.

**TABLE 2:** The measurement model and good fit values (*n* = 638).

Model	χ^2^	df	χ^2^/df	GFI	CFI	NFI	TLI	RMSEA	SRMR
Fit index values	121.301**	35	3.466	0.962	0.959	0.944	0.948	0.062	0.036
Acceptable fit values				0.900	0.900	0.900	0.900	0.080	0.050

**p < 0.01. GFI, goodness of fit index; CFI, comparative fit index; NFI, normed fit index; TLI, tucker-Lewis index; RMSA, root mean square error of approximation; SRMR, standardized root mean square residual.

### 3.3. Criterion-related validity analysis

This study used SWEMWBS, MHLQ, GWBS, LSS, Self-rated health, DASS and the Loneliness Scale to assess the criterion-related validity of MHPK-10-C. The results are shown in Table 3.

**TABLE 3:** Correlations between scales (*n* = 638).

	Mean ± SD	2	3	4	5	6	7	8
1. MHPL-10-C^+^	37.41 (6.85)	0.615**	0.349**	0.435**	0.479**	0.511**	−0.476**	−0.342**
2. SWEMWBS^#^	24.23 (4.48)		0.356**	0.651**	0.663**	0.660**	−0.603**	−0.444**
3. MHLQ^##^	38.78 (7.47)			0.243**	0.283**	0.290**	−0.300**	−0.198**
4. GWBS^++^	5.30 (1.13)				0.828**	0.613**	−0.499**	−0.436**
5. LSS^+^	5.09 (1.18)					0.636**	−0.528**	−0.413**
6. Self-rated health	3.69 (0.80)						−0.596**	−0.437**
7. DASS^§§^	33.16 (8.37)							0.499**
8. Loneliness	2.67 (0.82)							

**p < 0.01. SD, standard deviation.

^+^MHPL-10-C, positive mental health literacy scale (Chinese version).

^#^SWEMWBS, Warwick Edinburgh mental wellbeing scale (short version).

^##^MHLQ, mental health literacy questionnaire.

^++^GWBS, general wellbeing sale.

^+^LSS, the life satisfaction scale.

^§§^DASS, depression anxiety stress scale.

### 3.4. Reliability analysis

Cronbach’s alpha coefficient of the MHPK-10-C scale (*n* = 638) was 0.869; the intraclass correlation coefficient for the test–retest score was 0.721 (*p* < 0.01), and the test–retest reliability of each item was between 0.327 and 0.708 (see Table 4).

**TABLE 4:** Test–retest results–MHPK-10-C (*n* = 96).

Item	Pearson correlations
Item_1 Handling stressful situations in a good manner 1. 能妥善处理压力	0.499**
Item_2 Believing in yourself 2. 对自己有信心	0.708**
Item_3 Having good sleep routines 3. 有良好的睡眠习惯	0.578**
Item_4 Making decisions based on own will 4. 能根据自己的意愿做决定	0.475**
Item_5 Setting limits for your own actions 5. 会约束自己的行为	0.327**
Item_6 Feeling that you belong in a community 6. 有团体归属感	0.594**
Item_7 Mastering your own negative thoughts 7. 能控制自己的负面想法	0.366**
Item_8 Setting limits for what is OK for me 8. 能控制自己的喜好，如饮酒、上网有节制。	0.515**
Item_9 Feeling valuable regardless of your own accomplishments 9. 不论成就如何，都觉得自己有价值	0.402**
Item_10 Experiencing school mastery 10. 能游刃有余的处理学校各种事情	0.576**
Total MHPK-10-C	0.721**

**p < 0.01.

## 4. Discussion

### 4.1. Analysis of psychometric properties

After item analysis, it was found that the decision value of MHPK-10-C item’s extreme group comparison was greater than 3.000, and the correlation between items and the total score was more than 0.400 (see Table 1), indicating that each item was well differentiated. One potential limitation was the possibility of MHPK-10 having a ceiling effect (Mean = 4.51, SD = 0.54, Minimum = 0, Maximum = 5) as “The ceiling effect may cause difficulties in establishing the discriminant validity of the scale” (Bjørnsen et al., 2017). However, this effect was not apparent in the present study which is evident by the sample’s mean scores on the instrument (Mean = 3.75, SD = 0.70 Minimum = 0, Maximum = 5).

The results of exploratory factor analysis showed that 10 items in the MHPK-10-C were consistent with the original scale, and one factor was extracted. The commonality of all items was greater than 0.300, the load was greater than 0.400, and the cumulative variance contribution difference was greater than 40.00% (see Table 1). The results of confirmatory factor analysis showed that the one factor model fitted well, with GFI, CFI, NFI, and TLI higher than 0.900, and RMSEA and SRMR less than 0.080 (see Table 2), indicating that the model fitted well on the whole (Zhonglin et al., 2018).

Positive MHL is an important part of MHL (Kutcher et al., 2016). When evaluating the criterion validity of the MHPK-10-C, we believe that positive mental health (SWEMWBS, GWBS, LSS) and MHL (MHLQ) should be included. In addition, MHL is considered to be an important factor in promoting an individual’s mental health (Kutcher et al., 2015; Ming and Chen, 2020), and Bjørnsen found that MHL is closely related to mental health (Bjørnsen et al., 2017). Therefore, a neutral mental health scale (Self-rated health) or illness mental health scale (DAP, Loneliness) may also be selected. The results showed that the total score of MHPK-10-C was significantly positively correlated with SWEMWBS, MHLQ, GWBS, LSS, and Self-rated health, and significantly negatively correlated with the DASS and loneliness scales (see Table 3). Table 3 shows that MHPK-10-C is closely related to positive psychology, MHL and individual mental health, which is consistent with the findings of Bjørnsen et al. (2019) and Guimarães et al. (2022).

In terms of reliability, the Cronbach’s α of the MHPK-10-C was 0.869. A Cronbach’s alpha coefficient of 0.80 ≤ α ≤ 1.00 reflects high reliability (Özpulat et al., 2022) and Cronbach’s alpha of the original scale was 0.84 (Bjørnsen et al., 2017). Similarly,α was 0.79 for the Portuguese version (Guimarães et al., 2022) and 0.871 for the Turkish version (Özpulat et al., 2022).

The test–retest reliability was 0.721, and 0.74 for the original scale (Bjørnsen et al., 2017). Similarly, the test–retest reliability of each item of the revised Chinese version was between 0.327 and 0.708 (see Table 4), while that of the Portuguese version was between 0.28 and 0.79 (Guimarães et al., 2022). The test–retest reliability of individual items was relatively low, possibly because item responses may have been affected by an individuals’ situation at the time and can fluctuate. Nevertheless, the total test–retest reliability reached 0.721, which is acceptable (the critical value was 0.70) (Bjørnsen et al., 2017). In general, MHPK-10-C has sound reliability.

### 4.2. MHPK-10-C of Chinese teenagers

The results of this survey show that the average MHPK-10 score of Chinese teenagers was 3.74, while the scores of Norwegian, Portuguese and Turkish teenagers are 4.518 (Bjørnsen et al., 2017), 4.280 (Guimarães et al., 2022) and 2.900 (Özpulat et al., 2022), respectively. There are several possible reasons for the different scores. First, MHL is affected by a country’s economic performance. Norway and Portugal are both developed countries, while China and Türkiye are both developing countries and studies have shown that levels of MHL are higher in developed countries than in developing countries (Dang et al., 2018). Second, MHL is affected by the availability of local mental health resources. Ozpulat believes that the lower MHPK-10 scores of adolescents in Türkiye than those of adolescents in Norway may be because most adolescents in Türkiye do not receive mental health education or training. Alternatively, this may be related to an insufficient provision of local mental health resources in Türkiye (Özpulat et al., 2022). Like Türkiye, the mental health resources available to Chinese adolescents also need to be increased.

### 4.3. Strengths and limitations

The main advantages of this study were a sufficiently large sample size and a high response rate. The study has confirmed that MHPK-10-C has sound psychometric properties in terms of discrimination, reliability, and validity. In particular, in terms of differentiation, both the original scale (Bjørnsen et al., 2017) and the Portuguese version (Guimarães et al., 2022) were concerned about the scale having a ceiling effect which was not apparent in the present study. Our results also indicated that the positive MHL of Chinese adolescents should be enhanced. Fortunately, all entries in MHPK-10 are considered to be translatable into practice (Bjørnsen et al., 2017), which is likely to have a positive impact on the education of positive MHL.

Of course, there are also some limitations to this study. First, the subjects who participated in the study were from one junior college in Hunan Province, China, and most were women (76.18% of the total number). Therefore, our results may not be generalizable to other populations. Second, in order to facilitate the acquisition of responses by minimizing response time (Wu et al., 2020), the survey was conducted online (no paper survey), which may affect the quality of the survey results. However, some studies show that online surveys are influenced less by social expectations than face-to-face paper surveys (Velten et al., 2018).

The study authors believe that in order to address the deficiencies of the current research, follow-up research can be improved by: first, the survey should be expanded to include participants from different regions, and different ages (not only limited to teenagers). The second is to adopt a combination of online surveys and paper questionnaires.

## 5. Conclusion

The purpose of this study was to revise the MHPK-10 and analyze its psychometric characteristics. The study found that the MHPK-10-C has sound reliability and validity within the context of Chinese culture and can be used to measure positive MHL in Chinese adolescents. Compared to the ceiling effect of MHPK-10, MHPK-10-C has a better discrimination, which indirectly indicates that the level of positive MHL of Chinese adolescents is lower than that of adolescents from developed countries such as Norway and Portugal.

## Data availability statement

The original contributions presented in this study are included in the article/supplementary material, further inquiries can be directed to the corresponding author.

## Ethics statement

This study was approved by the Ethics Committee of the School of Education at Guangzhou University (IRB number: GZHU2020010). Written informed consent to participate in this study was provided by the participants’ legal guardian/next of kin.

## Author contributions

ZL and JD contributed to conception and design of the study. FY organized the database. JZ performed the statistical analysis. ZL wrote the first draft of the manuscript. ZL, FY, JZ, and JD wrote sections of the manuscript. All authors contributed to manuscript revision, read, and approved the submitted version.

## Footnotes

1
https://www.wjx.cn/
